# Deletion of murine *Arv1* results in a lean phenotype with increased energy expenditure

**DOI:** 10.1038/nutd.2015.32

**Published:** 2015-10-19

**Authors:** W R Lagor, F Tong, K E Jarrett, W Lin, D M Conlon, M Smith, M Y Wang, B O Yenilmez, M G McCoy, D W Fields, S M O'Neill, R Gupta, A Kumaravel, V Redon, R S Ahima, S L Sturley, J T Billheimer, D J Rader

**Affiliations:** 1 Department of Molecular Physiology and Biophysics, Baylor College of Medicine, Houston, TX, USA; 2 Division of Translational Medicine and Human Genetics, Perelman School of Medicine, University of Pennsylvania, Philadelphia, PA, USA; 3 Division of Endocrinology, Diabetes and Metabolism, Perelman School of Medicine, University of Pennsylvania, Philadelphia, PA, USA; 4 Department of Pediatrics, Columbia University Medical Center, New York, NY, USA

## Abstract

**Background:**

ACAT-related enzyme 2 required for viability 1 (*ARV1*) is a putative lipid transporter of the endoplasmic reticulum that is conserved across eukaryotic species. The ARV1 protein contains a conserved N-terminal cytosolic zinc ribbon motif known as the ARV1 homology domain, followed by multiple transmembrane regions anchoring it in the ER. Deletion of *ARV1* in yeast results in defective sterol trafficking, aberrant lipid synthesis, ER stress, membrane disorganization and hypersensitivity to fatty acids (FAs). We sought to investigate the role of Arv1 in mammalian lipid metabolism.

**Methods:**

Homologous recombination was used to disrupt the *Arv1* gene in mice. Animals were examined for alterations in lipid and lipoprotein levels, body weight, body composition, glucose tolerance and energy expenditure.

**Results:**

Global loss of *Arv1* significantly decreased total cholesterol and high-density lipoprotein cholesterol levels in the plasma. *Arv1* knockout mice exhibited a dramatic lean phenotype, with major reductions in white adipose tissue (WAT) mass and body weight on a chow diet. This loss of WAT is accompanied by improved glucose tolerance, higher adiponectin levels, increased energy expenditure and greater rates of whole-body FA oxidation.

**Conclusions:**

This work identifies *Arv1* as an important player in mammalian lipid metabolism and whole-body energy homeostasis.

## Introduction

ACAT (Acyl-CoA cholesterol acyl transferase) related enzyme 2 required for viability 1 (*ARV1*) is a transmembrane protein of the endoplasmic reticulum (ER) that is conserved between plants, yeast and mammals.^1, 2^ *ARV1* was originally identified in a screen for genes required for viability in the absence of *ARE1* and *ARE2*—the yeast homologs of Acyl-CoA cholesterol acyl transferase 1 and 2.^2^ Yeast deficient in *ARV1* accumulate sterols in the ER, and show reduced sterol content in the plasma membrane based on biochemical membrane fractionation,^2^ as well as filipin staining.^3, 4, 5, 6^ Concomitant with ER lipid retention, yeast lacking *ARV1* also harbor major defects in phospholipid, sphingolipid^7^ and glycosylphosphatidylinositol synthesis,^4^ and are particularly susceptible to sterol-binding antibiotics such as nystatin^2, 8^ and amphotericin B.^6^ Lipid accumulation in the ER in *ARV1*-deficient yeast is accompanied by severe ER stress and activation of the unfolded protein response.^5^ Most recently, Ruggles *et al.* performed a genome-wide screen for palmitoleate-induced fatty acid (FA) toxicity in yeast.^9^ Yeast strains deleted for *ARV1* are viable but highly sensitive to unsaturated FAs. In this same study, overexpression of human *ARV1* increased lipid synthesis in HEK293 cells and mouse liver, whereas knockdown of *Arv1* in MIN6 pancreatic β-cells and HEK293 cells inhibited lipogenesis and caused lipoapoptosis.^9^

**Figure 1:**
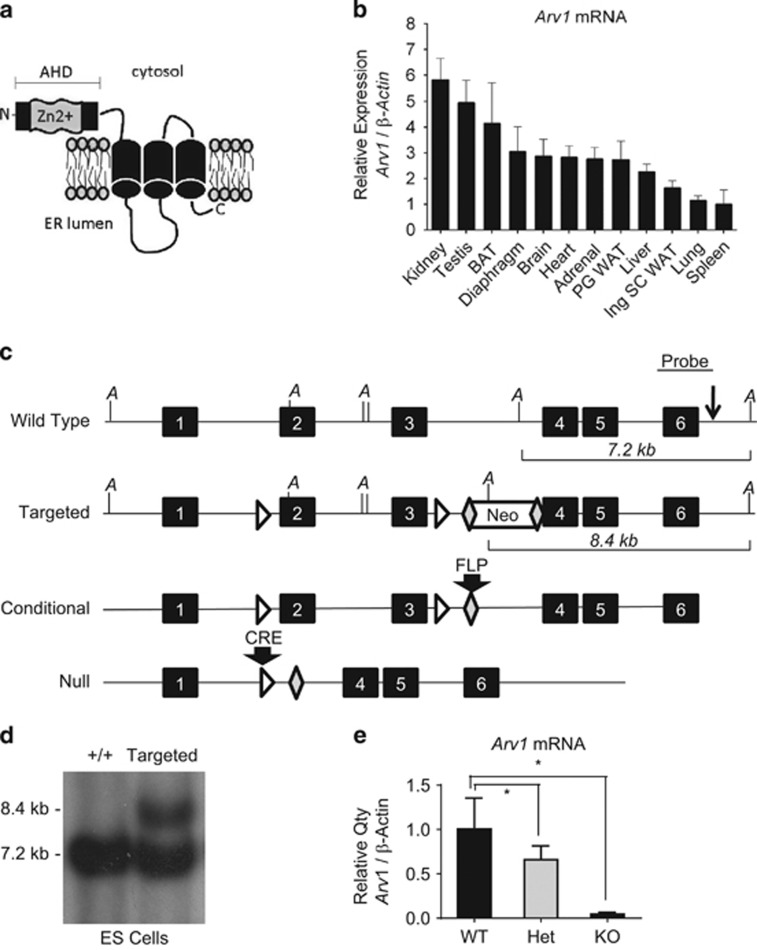
Generation of *Arv1* knockout mice. (**a**) Predicted structure of the Arv1 protein showing the N-terminal Zn^2+^ binding domain, Arv1 homology domain (AHD), transmembrane domains (barrels) and luminal C-terminus. (**b**) *Arv1* messenger RNA (mRNA) expression in tissues from male C57BL6/J mice 12–14 weeks of age (*n*=4), fold changes are shown relative to spleen, the lowest expression tissue. (**c**) Diagram of the murine *Arv1* locus showing the locations of the PGK-neomycin resistance cassette (‘Neo'), *LoxP* sites (triangles), FRT sites (diamonds), Exons (closed boxes), AseI restriction sites (‘A'), and Southern blotting probe (probe). (**d**) Southern blot of wild-type (lane 1) and targeted (lane 2) embryonic stem cells. Digestion of the wild-type *Arv1* allele with AseI produces a 7.2 kb fragment, while inclusion of the Neo cassette removes this AseI site and introduces a new site closer to exon 3, generating an 8.4 kb fragment. (**e**) *Arv1* mRNA levels measured by real-time reverse transcriptase PCR (RT-PCR) of liver RNA isolated from wild-type (WT), heterozygous (Het) and homozygous null mice (KO) generated using CMV-*Cre* to delete *Arv1* in the germline. Values are expressed as means±s.d. where the asterisk (*) indicates *P*<0.05 relative to the wild-type mice. CMV, cytomegalovirus.

The ARV1 protein contains a conserved N-terminal zinc ribbon motif known as the ‘*Arv1* homology domain,' (AHD) which is followed by several transmembrane regions. The predicted and experimentally defined topology of the yeast Arv1p indicated the AHD is cytosolic. This is followed by a transmembrane region, a large loop region located inside the ER lumen, and two more transmembrane domains with the extreme C-terminus of the protein extending back into the ER lumen^10^ (Figure 1a). The murine *Arv1* gene encodes a 266 amino acid (AA) protein. In contrast to its 321 AA yeast counterpart, murine *Arv1* differs in that the AHD is preceded by a N-terminal 28 amino acid leader sequence (5–6). Expression of human *ARV1* functionally complements the loss of *ARV1* in yeast, rescuing all of the known growth and viability defects.^2, 7^ Despite this functional conservation, very little is known about the biochemical activity of *Arv1* in any organism. In the current study, we sought to test the involvement of *Arv1* in mammalian lipid metabolism by generating mice with a germline deletion of the gene. To our surprise, *Arv1* knockout (*Arv1* KO) mice have a striking metabolic phenotype including major reductions in white adipose tissue (WAT) mass, improved glucose tolerance and increased energy expenditure. These findings are consistent with an important role for *Arv1* in FA homeostasis, and identify it as a novel regulator of body composition and energy expenditure in mice.

## Materials and methods

### Generation of *Arv1* knockout mice

A genomic library derived from female 129S6/SvEvTac (Taconic, Hudson, NY, USA) mice was screened with a cDNA probe corresponding to the *Arv1* gene to identify positive BAC clones. Two BAC clones were used to generate the targeting vector illustrated in Figure 1. Positive embryonic stem cell clones (R1 cells, SCRC-1011, American Type Culture Collection, Manassas, VA, USA) were selected with G418, and genomic integration was confirmed by Southern blotting with an AseI digest. Mycoplasma testing was not performed on embryonic stem cells before injection. Correctly targeted embryonic stem cells were injected into blastocysts from C57Bl/6J mice. Male chimeras were bred to C57BL6/J mice to obtain F1 progeny. These mice were then crossed with FLP recombinase transgenic mice (C57BL6/J) to remove the neomycin resistance gene, creating a conditional *Arv1* allele with exons 2 and 3 flanked by *loxP* sites. The mice harboring the conditional *Arv1* allele were then crossed with CMV-*Cre* transgenic mice on a C57BL6/J background from Jackson Labs to delete *Arv1* in the germline (B6.C-Tg(CMV-cre)1Cgn/J, Jax stock number 006054), and backcrossed an additional two to four times before experiments.

### Genotyping

The *Cre* transgene was detected by PCR using published primers—*Cre-For:* 5′-ACGACCAAGTGACAGCAATG-3′, *Cre-Rev:* 5′-CTCGACCAGTTTAGTTACCC-3′ 350 bp.^11^ Primers from Taconic for mouse chromosome 11 were included as a positive control (1260_1: 5′-GAGACTCTGGCTACTCATCC-3′, 1260_2: 5′-CCTTCAGCAAGAGCTGGGGAC-3′ 585 bp). The WT *Arv1* allele was detected using the primers *WTArv1For:* 5′-CTTTGTTATGTTGTACAGGGTTAC-3′, *WTArv1Rev:* 5′-CCCGTGGACATGGACATGGGAAC-3′ 218 bp, where the forward primer binding site is unique to the WT allele in Intron 1. The floxed *Arv1* allele was detected using the primers *FlxFor*: 5′-GCAGCCCAATTCCGATCATATTC-3′, *FlxRev*: 5′-CAGGAACAGAAATCACAGGCAG-3′, to amplify a 162 bp fragment of intron 1 where the forward primer binds to the *loxP* site. In the reactions to detect the WT and floxed alleles, primers that detect the murine IL2 genomic sequence from JAX were included as a positive control (*JAX-oIMR7338*: 5′-CTAGGCCACAGAATTGAAAGATCT-3′, *JAX-oIMR7339*: 5′-GTAGGTGGAAATTCTAGCATCATCC-3′ 324 bp). The deleted (null) *Arv1* allele was detected with the primers: *GENO3WTARV1F*: 5′-CATTTGAGAGGGTAGCACAACTAC-3′, *GENO-WTARV1-Rev*: 5′-GACTTCCTCTCAGACTCCTCTACCGTGAG-3′ and *GENO3DELARV1R*: 5′-CCTGGCTTAAGGCAAACGCTTCTGAAG-3′. The first two primers flank the *loxP* site in intron 1, while the third primer binds to intron 3, which is brought close enough for amplification when the allele is deleted (Floxed 409 bp, WT 391 bp, null 182 bp). Annealing temperatures for PCR were as follows: *Cre*—60 ºC, WT—58 ºC, Floxed—58 ºC, Null—55 ºC. To detect the WT allele, a 2:1 molar ratio of target primers to internal control primers was optimal.

### Animal care and treatment

All mice were housed in a pathogen-free animal facility with a daylight cycle from 0700 to 1900 h. Animals were allowed free access to food and water, and were maintained on a standard rodent chow diet (Purina 5010). One cohort of animals were fed a western-type high-fat diet containing 0.2% cholesterol and 45% kcal per fat (Research Diets D12079B) from weaning (Supplementary Data). Non-fasted body weights were measured once weekly on the weekday of birth beginning at the time of weaning. Fasting for lipid and glucose phenotyping was performed for 4 h to allow for clearance of intestinally derived triglycerides (TGs). Blood glucose was measured via the tail vein with the OneTouch Ultra glucometer following a 4-h fast (Lifescan; Johnson & Johnson, Milpitas, CA, USA). Immediately after tail glucose measurement, plasma was obtained for lipid analysis by retro-orbital bleeding with heparinized natelson collecting tubes under the influence of isofluorane anesthesia. Plasma insulin levels were measured with the Ultra Sensitive Mouse Insulin ELISA (Crystal Chem, Downers Grove, IL, USA).^12^ All the procedures were performed according to the regulations and with the prior approval of the Institutional Animal Care and Use Committees of the University of Pennsylvania (protocol 704534) and Baylor College of Medicine (protocol AN-6243).

### RNA isolation and real-time reverse transcriptase PCR

Total RNA was isolated from liver using TriZol (Invitrogen, Grand Island, NY, USA). The QIAGEN RNeasy mini kit was used to isolate RNA from nonhepatic tissues, with the exception of adipose and brain, in which cases the QIAGEN RNeasy lipid tissue mini kit proved superior. RNA (1 μg) was reverse transcribed into cDNA using the SuperScript III Kit (Invitrogen) or the iScript kit (Bio-Rad, Hercules, CA, USA). The cDNA products were diluted 1:20 into a final reaction volume of 12.5 μl in the wells of a 384-well reaction plate containing 300 nm of each forward and reverse primer, and 6.25 μl of 2X SYBR green master mix (Applied Biosystems, Grand Island, CA, USA). The following primers were used to amplify exons 2 and 3 of mouse ARV1: forward 5′-TCAGGAGCTGTACCGGGACTA-3′, reverse 5′-CTGCAGCTGCCACCACCGCAGGTATGCTTCA-3′. Beta actin was amplified with the following primers: forward 5′-TTGGGTATGGAATCCTGTGG-3′, reverse 5′-CTTCTGCATCCTGTCAGCAA-3′. A Taqman assay to exons 2 and 3 was also used to measure *Arv1* expression (Mm01253489_m1, Applied Biosystems) with Premix Ex Taq (RR390L, Clontech, CA, USA). Relative quantities were calculated using the delta delta Ct method.^13^

### Histology

WAT and brown adipose tissue (BAT) were harvested and fixed in 10% buffered formalin overnight, dehydrated in a series of ethanol washes and embedded in paraffin. Tissue sections were stained with the hematoxylin and eosin by the Penn Cardiovascular Institute Histology Core and the Texas Medical Center—Digestive Diseases Center, Cellular Morphology Core.

### Lipid measurements

Plasma total and high-density lipoprotein cholesterol (HDL-C) were measured enzymatically on a Cobas MIRA II (Roche Diagnostic Systems, Indianapolis, IN, USA) using reagents from Diagnostic Chemicals Limited. Plasma adiponectin levels were measured using mouse adiponectin ELISA kits from Millipore Corporation (Billerica, MA, USA). Hepatic TG levels were measured by homogenizing liver in three volumes of phosphate-buffered saline. The liver lysates were diluted 1:5 in phosphate-buffered saline, and 20 μl was transferred to the well of a microplate. TGs were solubilized with the addition of 20 μl of 1% deoxycholate to each well for 5 min at 37 °C. TGs were then measured using the Infinity triglyceride reagent (Thermo, Grand Island, NY, USA).

### Glucose tolerance tests

Intraperitoneal glucose tolerance tests were performed on mice fasted overnight (16 h). Mice were given an intraperitoneal injection of 2 g glucose per kg body weight (BW), and blood glucose levels were measured at 0, 15, 30, 60, 90 and 120 min later via the tail vein.

### Metabolic phenotyping and activity measurements

Body composition was measured by DEXA (dual-energy X-ray absorptiometry) scan and magnetic resonance imaging by the Mouse Phenotyping, Physiology and Metabolism Core at the University of Pennsylvania School of Medicine as previously described.^14^ Metabolic rates were measured by indirect calorimetry in open-circuit oxymax chambers that are a component of the Comprehensive Lab Animal Monitoring System (Columbus Instruments, Columbus, OH, USA). Mice were housed singly and maintained at 24 °C under a 12-h light–dark cycle (light period 0700–1900 h). Food and water were available *ad libitum*. All the mice were acclimated to monitoring cages for 24 h before beginning the physiological recordings. To calculate oxygen consumption (VO_2_), carbon dioxide production (VCO_2_) and RER (ratio of VCO_2_ to VO_2_), gas concentrations were measured at the inlet and outlet of the sealed chambers. VCO_2_, VO_2_ and calculated heat were normalized to lean mass determined by magnetic resonance imaging scan for each animal.

### Oral fat tolerance and whole-body FA oxidation

Female mice were fasted for 14 h before oral fat tolerance testing. Mice received an oral gavage of 15 μl of olive oil per gram of body weight. Plasma was collected via retro-orbital bleeding under anesthesia before gavage, and then at 1, 3, 5 and 7 h later. Plasma TGs were measured by using the Infinity Triglycerides enzymatic assay kit from Thermo Fisher (Grand Island, NY, USA), using human control serum as a standard from 0 to 30 μg per well (CS1 human serum, Wako Diagnostics, VA, USA). Whole-body FA oxidation was measured on the basis of previously published methods^15, 16^ by the Mouse Metabolic Phenotyping Core at Penn. Briefly, mice were fasted 24 h before the experiment, injected intraperitoneally with 500 μl of [3H]-oleic acid (10 μCi, 2 mm oleic acid: 2% BSA) and killed exactly 30 min later for plasma. An aliquot of plasma (15 μl) was transferred to a new tube and then diluted with 50 μl of 10% BSA and 0.5 ml water. Next, 50 μl of perchloric acid (HClO_4_) were added to the sample to precipitate albumin-bound FAs. The aqueous phase (containing [3H]-H_2_O resulting from FA oxidation) was measured by scintillation counting. The counts were corrected for the specific activity of the total [3H] in the plasma and normalized to the fasting body weight of each animal, reported as microgram oleate oxidized per kilogram body weight per hour.

### Statistical analyses

Animal numbers were estimated on the basis of previous experience with each specific assay and expected effect size. As comparisons were made across genotypes, no pre-randomization was performed. Researchers were not blinded to genotype. Animals were sex-matched and age-matched for all the experiments as detailed in the figure legends. Data were normally distributed and similar variances were observed in both the groups. All the data are presented as means±s.d. Differences between the two groups were assessed with a two-tailed, unpaired Student's *t*-test. Comparisons involving multiple groups were tested by one-way analysis of variance and Tukey's post test. *P*-values <0.05 were considered statistically significant (*).

## Results

To test the role of Arv1 in mammalian lipid metabolism, we first examined *Arv1* RNA expression in mouse tissues. *Arv1* messenger RNA was present at a low level in all the tissues with greatest expression observed in the kidney, testes and BAT (Figure 1b, Supplementary Figure 1). We next used gene targeting to generate mice with a global deletion of *Arv1* (Figures 1c and d). Loss of critical exons 2 and 3 produces a nonfunctional message, which encodes the first 53 AAs of the 266 AA ARV1 protein followed by 18 novel frameshifted AAs and a premature stop codon. This truncated protein fragment (53/266 total AAs) would lack the last cysteine residue in the putative zinc ribbon motif^6^ and all transmembrane domains. Liver *Arv1* messenger RNA levels were reduced by >35% in the heterozygous and 95% in the homozygous knockout mice (Figure 1e, *P*<0.05). *Arv1* KO mice were born in the expected Mendelian ratio. Female KO mice were found to be infertile, so mice for experiments were generated by breeding heterozygotes.

**Figure 2:**
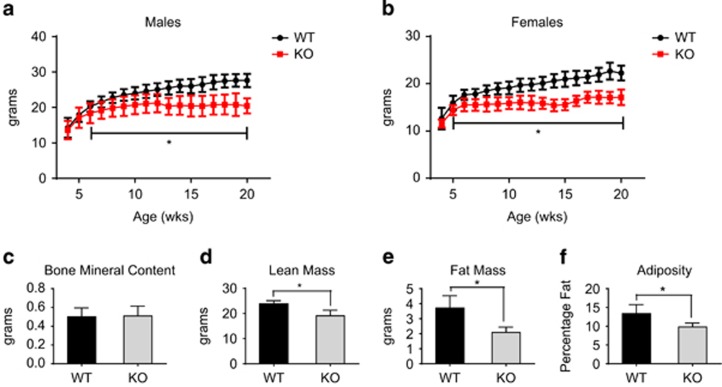
*Arv1*-deficient mice have reduced body weight and decreased fat mass on a chow diet. Growth curves of (WT) and *Arv1* knockout (KO) mice on a chow diet for (**a**) males (WT *n*=19, KO *n*=16) and (**b**) females (WT *n*=15, KO *n*=8). Body composition was examined in chow-fed male mice at 4–6 months of age by DEXA scan (WT *n*=6, KO *n*=6); (**c**) bone mineral content, (**d**) lean mass, (**e**) fat mass and (**f**) adiposity were determined. All the values are reported as the mean±s.d. (*P<0.05).

Wild-type (WT) and *Arv1* KO mice on a chow diet were weighed weekly to generate growth curves (Figures 2a and b). *Arv1* KO mice were not significantly lighter than WT animals at the time of weaning, but failed to gain body weight in adulthood (>8 weeks of age), reaching an average maximum of around 17 g for females and 20 g for males (similar results were also observed on a western-type high-fat diet; Supplementary Figure 2). *Arv1* KO mice showed substantial reductions in subcutaneous WAT and perigonadal WAT relative to WT mice at 12 weeks of age. In contrast, BAT, liver, heart, lungs, spleen, kidney and brain weights were not significantly different from WT animals (Supplementary Figure 3). To gain further insight into the difference in body weight, we examined 4–6-month-old male *Arv1* KO mice by DEXA scan. Male *Arv1* KO mice had body lengths identical to those of WT mice, whereas females were modestly shorter (Supplementary Figures 3j and 4j). Total bone mineral content was also not different (Figure 2c), indicating that alterations in the bone size or density were not major contributors to the difference in body weight. *Arv1* KO mice showed significant reductions in lean mass (20%, *P*=0.001), as well as fat mass (44%, *P*=0.001; Figures 2d and e). The decrement in fat mass was greater than the difference in lean mass, and the animals consequently had a much lower percentage of total body fat (adiposity; Figure 2f). We also noticed that *Arv1* KO mice appeared to be more susceptible to spontaneous death in adulthood (3–6 months of age) than WT. This effect was not completely penetrant- manifest in ~30% of the mice by 5 months of age (Supplementary Figure 5). The *Arv1* KO mice also did not cope well with overnight fasting, which was required in some of the subsequent experiments (12–16 h), with a fraction of them (approximately one out of six mice) appearing cold to the touch, hypoglycemic and moribund the next morning.

**Figure 3:**
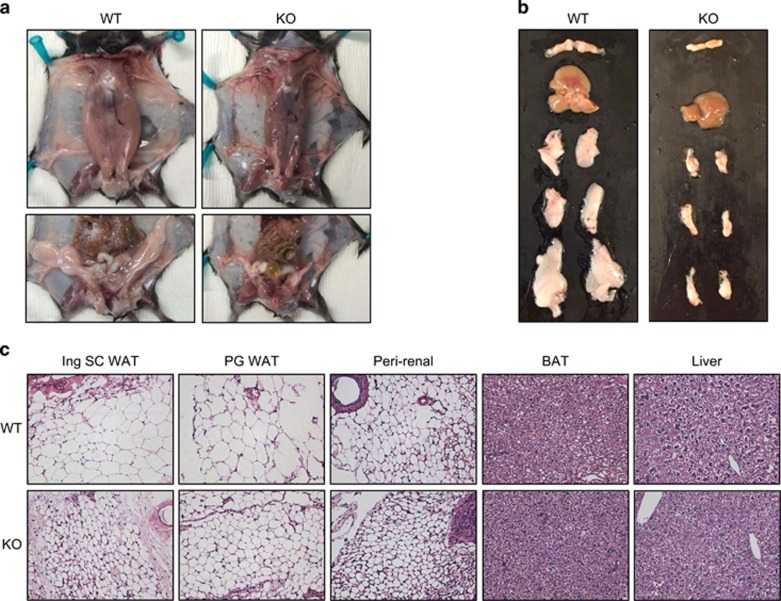
Abnormal white adipose tissue morphology in *Arv1* KO mice. (**a**) Gross appearance of littermate male WT and *Arv1* KO mice at 6 months of age showing lack of subcutaneous (SC WAT) and perigonadal (PG WAT) white adipose tissue. (**b**) Image of brown adipose tissue (BAT), liver, axillo–thoracic subcutaneous white adipose tissue (axillo–thoracic SC WAT), inguinal subcutaneous white adipose tissue (Ing SC WAT) and perigonadal white adipose tissue (PG WAT). (**c**) Histological appearance of hematoxylin and eosin stained subcutaneous inguinal subcutaneous WAT (Ing SC WAT), perigonadal WAT (PG WAT), perirenal WAT (PR WAT), brown fat (BAT) and liver at × 400 magnification.

WAT was noticeably depleted in *Arv1* KO mice (Figure 3a, Supplementary Figure 6) with very little subcutaneous or perigonadal WAT obtained upon dissection. In contrast, livers and BAT pads appeared grossly normal (Figure 3b). At a cellular level, hematoxylin and eosin staining of paraffin sections revealed markedly different white adipose morphology. *Arv1* KO adipose exhibited more intense cytoplasmic staining and dramatically smaller white adipocytes in all of the depots examined—namely inguinal subcutaneous, perigonadal, mesenteric and perirenal WAT (Figure 3c).

**Figure 4:**
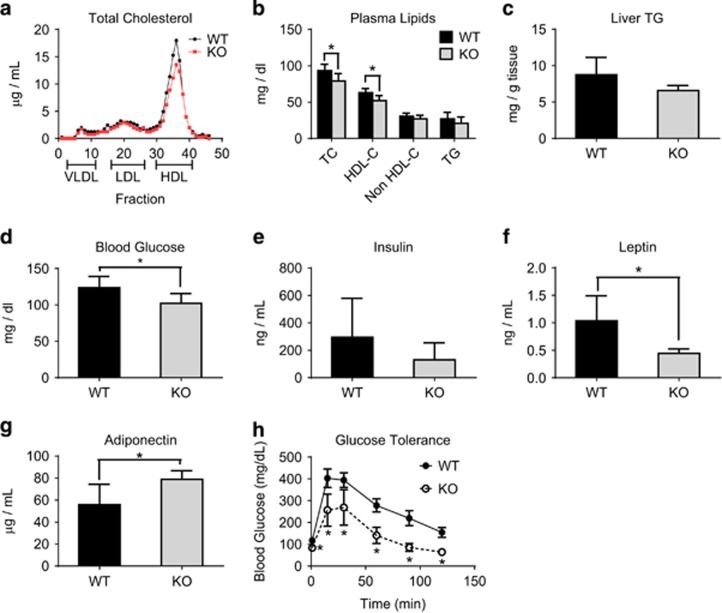
Plasma lipids, glucose and adipokine levels in *Arv1* KO mice. (**a**) Total cholesterol in lipoprotein fractions of pooled plasma from 10-week-old male WT and *Arv1* KO mice fasted 4 h. (**b**) Fasting plasma lipid levels (4 h) were determined in chow-fed male WT (*n*=12) and *Arv1* KO mice (*n*=9). (**c**) Liver triglycerides were measured in male WT (*n*=9) and *Arv1* KO mice (*n*=3) at 12 weeks of age. (**d**) Fasting blood glucose (4 h) in male WT (*n*=11) and *Arv1* KO mice (*n*=9). (**e**) Plasma insulin (WT *n*=6, KO *n*=5), (**f**) leptin (WT *n*=9, KO *n*=6) and (**g**) adiponectin (WT *n*=9, KO *n*=6) levels in male mice after a 4 h fast at 3–5 months of age. (**h**) Intraperitoneal glucose tolerance test was performed in male WT and *Arv1* KO mice following an overnight (16 h) fast (WT *n*=6, KO *n*=6). All the values are reported as the mean±s.d. (*P<0.05).

*Arv1* KO mice had significantly lower plasma lipids. Separation of plasma lipoproteins by gel filtration chromatography revealed a difference in the HDL-C peak (Figure 4a, Supplementary Figure 7). Male *Arv1* KO mice had 15% lower total cholesterol (*P*=0.003) and 17% lower HDL-C (*P*=0.0008), whereas non HDL-C and TGs were not significantly different between the groups (Figure 4b). Despite the major reductions in WAT mass, we did not observe an increase in the hepatic TG content (Figure 4c). No difference was observed in fasting glucose in female mice (Supplementary Figure 7), however, male *Arv1* KO mice had lower blood glucose after a 4 h fast (Figure 4d), and plasma insulin levels were not significantly different. (Figure 4e). Consistent with decreased WAT mass, *Arv1* KO mice had significantly reduced leptin (Figure 4f), but interestingly had increased plasma adiponectin relative to WT controls (Figure 4g). Male *Arv1* KO mice had a dramatic improvement in glucose tolerance compared with WT animals (AUC reduced by 45%, Figure 4h).

**Figure 5:**
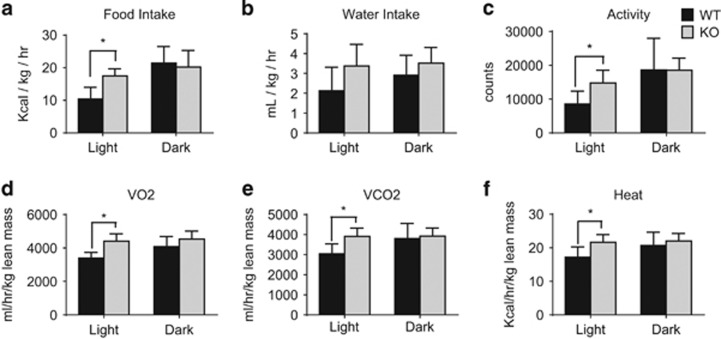
*Arv1* knockout mice have increased energy expenditure. Metabolic parameters and activity were measured over a 24 h period in 4–6-month-old male WT (*n*=6) and *Arv1* KO (*n*=5) mice on a chow diet. (**a**) Food consumption, (**b**) water intake, (**c**) activity, (**d**) oxygen consumption (VO2) normalized to lean mass, (**e**) carbon dioxide production (VCO2) normalized to lean mass and (**f**) calculated heat production normalized to lean mass are shown. All the values are reported as the mean±s.d. (*P<0.05).

**Figure 6:**
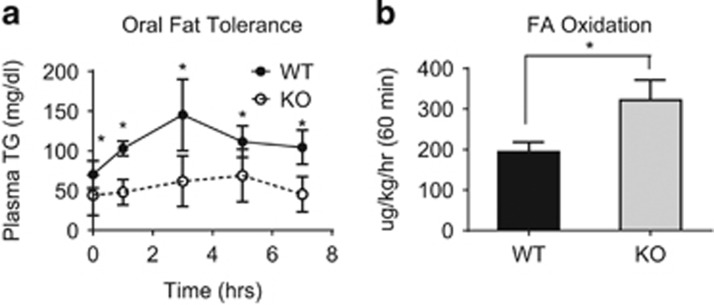
(**a**) Oral fat tolerance was measured after a bolus of olive oil in female WT (*n*=7) and *Arv1* KO (*n*=6) mice. (**b**) Whole-body oxidation of [3H]-oleic acid was measured in female WT (*n*=4) and *Arv1* KO (*n*=5) following a 24-h fast. All the values are reported as the mean±s.d. (*P<0.05).

The *Arv1* KO mice were examined using metabolic cages to investigate the basis for the decreased fat mass and body weight. *Arv1* KO mice consumed more food during the light cycle than WT mice (Figure 5a). No differences were observed in water intake (Figure 5b). Consistent with the increase in food consumption during the light hours, *Arv1* KO mice were also more active during the light period than WT animals (Figure 5c). As expected based on the reduced WAT mass, we found that *Arv1* KO animals did have increased oxygen consumption (VO_2_) and carbon dioxide production (VCO_2_), which reached statistical significance during the light hours (Figures 5d and e). The respiratory exchange ratio (RER) did not vary by genotype in the light phase, and the KO was only modestly lower than WT in the dark phase (WT: 0.905±0.025 versus KO 0.864±0.019, −4.5%, *P*<0.05), suggesting there was no major shift in carbohydrate versus fat utilization. *Arv1* KO mice also expended more energy (calculated heat) than WT controls during the light cycle (Figure 5f). We hypothesized that the lack of TG stores in the WAT may be related to increased FA clearance and disposal. To test this, we measured fat tolerance in female *Arv1* KO mice after an oral bolus of olive oil. *Arv1* KO mice started out with a lower basal plasma TG level following an overnight fast, and had a nearly flat TG excursion after the olive oil gavage (Figure 6a). To test whether an increased rate of FA disposal could explain the difference in oral fat tolerance, we measured whole-body oxidation of a [3H]-oleic acid tracer. Consistent with the increase in energy expenditure, *Arv1* KO mice also showed a robust 66% elevation in the rate of FA oxidation (Figure 6b).

## Discussion

Mice lacking *Arv1* exhibit a dramatic lean phenotype with severely reduced WAT stores in all depots on a chow diet. We focused on male mice for the majority of studies, but it should also be noted that female mice lacking *Arv1* also had similarly reduced body weight, fat mass, plasma lipids and higher adiponectin (Supplementary Figure 4). The lean phenotype included increased energy expenditure, improved oral fat tolerance and a faster rate of whole-body FA oxidation. *Arv1* KO mice actually consumed more food (Figure 5a, Supplementary Figure 8) and were more active than WT mice (Figure 5c). Standard animal housing conditions are below thermoneutrality for mice, and in this setting spontaneous cage activity is generally not a major contributor to the differences in energy expenditure.^17^ However, further work is needed to determine the contributions of activity, nonshivering thermogenesis, and basal metabolic rate to the increased energy expenditure.

The global decrease in WAT in the *Arv1* KO mice is best described as a lean phenotype, as it was not accompanied by other features of congenital lipodystrophy. The best examples for comparison are the *Bscl2* KO,^18, 19, 20^ fatty liver dystrophy^21^ (*Fld*), *Agpat2* KO^22^ and A-ZIP^23^ transgenic mice. In all the cases, cell autonomous defects in adipocyte differentiation, viability or function prevent effective storage of FA as TG, and impair adipokine secretion. Without functional adipose tissue to sequester excess FA, severe insulin resistance, fatty liver and splenomegaly ensue. In contrast, *Arv1* KO mice have improved glucose tolerance, increased adiponectin, normal liver TG content and a complete lack of ectopic lipid deposition in other tissues. The *Arv1* KO mice have comparatively more adipose than these lipodystrophic mice (−44% subcutaneous WAT mass, −50% perigonadal WAT relative to WT controls) as well as normal BAT mass (Figure 2e). This suggests that adipocytes and fat reserves in the *Arv1* KO mice are smaller, but fundamentally healthy, and the global loss of fat mass is more likely owing to *Arv1* loss in other tissues.

Mutations in *ARV1* were initially identified as a consequence of inviability in the absence of sterol esterification in yeast. Beyond this context, loss of *ARV1* in any organism does indeed compromise growth but is not lethal. *ARV1* KO mice of both sexes appeared to be more susceptible to spontaneous death in adulthood (3–6 months of age) for reasons that are unclear. It is possible that *ARV1* KO mice are less able to cope with fluctuations in food availability without the buffer of additional TG stores in WAT. The lack of WAT TG stores for adequate insulation and thermogenesis could be a factor in their increased mortality. Although improved glucose tolerance is generally considered beneficial, a major defect in glucose production or mobilization may be detrimental in this case. In accordance with observations in yeast where *ARV1*-deficient cells were mating deficient, we observed that *Arv1* KO animals, particularly females, also had reproductive impairments. It remains to be determined whether these phenotypes reflect localized variation in sterol or FA esterification.

In earlier studies, we used antisense oligonucleotides (ASOs) to transiently knock down *Arv1* in adult C57BL6/J male mice.^24^ ASOs primarily targeted the liver, although significant knockdown of *Arv1* was also observed in adipose and intestine. ASO-mediated knockdown of *Arv1* resulted in a doubling of plasma cholesterol, a decrease in liver TG content, an increase in liver:body weight ratio, higher bile acid concentrations in the plasma and elevated transaminase levels (ALT). These lipid changes were accompanied by activation of the FXR regulatory pathway in the liver. In contrast, mice with a germline deletion of *Arv1* described in the current manuscript had reduced total and HDL-cholesterol (24% and 27% respectively, Figure 4b), unchanged liver:body weight ratios, and normal liver morphology and TG content. It is possible that the ASOs may have toxic and/or off-target^25, 26, 27, 28^ effects, or that a germline deletion may be distinct from the transient downregulation achieved with ASOs. The current data with whole-body deletion suggests an overwhelming and dominant role of ARV1 in extrahepatic tissues regulating energy metabolism; perhaps the brain, skeletal muscle or adipose—which merit further exploration.

A unifying model for these results may reflect a role for ARV1 in FA homeostasis. The ER is the site of FA ligation to CoA, as well as subsequent esterification in cholesteryl esters and TGs. ARV1 may be directly involved in lipid synthesis or storage, or may indirectly alter these processes through changes in ER lipid content. Indeed, in the yeast model of *ARV1* deficiency, we observed a severe UFA-sensitivity; among 152 strains that were hypersensitive to 0.5 mm palmitoleate loading, ARV1-deficient yeast were the most liposensitive.^9^ Studying ARV1 in isolated mammalian cell culture systems derived from the *Arv1* KO mice will shed light on these important questions. Further work is needed to define the physiological function of ARV1 in energy homeostasis by refining its site of action to specific tissues, such as the central nervous system, skeletal muscle and adipose. A better understanding of ARV1 and its downstream effectors may identify new targets for the treatment of obesity and insulin resistance in humans.

## Supplementary Material

Supplementary Informations

## References

[bib1] 1Fores O, Arro M, Pahissa A, Ferrero S, Germann M, Stukey J et al. *Arabidopsis thaliana* expresses two functional isoforms of Arvp, a protein involved in the regulation of cellular lipid homeostasis. Biochim Biophys Acta 2006; 1761: 725–735. 16725371 10.1016/j.bbalip.2006.03.025

[bib2] 2Tinkelenberg AH, Liu Y, Alcantara F, Khan S, Guo Z, Bard M et al. Mutations in yeast ARV1 alter intracellular sterol distribution and are complemented by human ARV1. J Biol Chem 2000; 275: 40667–40670. 11063737 10.1074/jbc.C000710200

[bib3] 3Villasmil ML, Ansbach A, Nickels JT Jr. The putative lipid transporter, Arv1, is required for activating pheromone-induced MAP kinase signaling in *Saccharomyces cerevisiae*. Genetics 2011; 187: 455–465. 21098723 10.1534/genetics.110.120725 PMC3030489

[bib4] 4Kajiwara K, Watanabe R, Pichler H, Ihara K, Murakami S, Riezman H et al. Yeast ARV1 is required for efficient delivery of an early GPI intermediate to the first mannosyltransferase during GPI assembly and controls lipid flow from the endoplasmic reticulum. Mol Biol Cell 2008; 19: 2069–2082. 18287539 10.1091/mbc.E07-08-0740 PMC2366835

[bib5] 5Shechtman CF, Henneberry AL, Seimon TA, Tinkelenberg AH, Wilcox LJ, Lee E et al. Loss of subcellular lipid transport due to ARV1 deficiency disrupts organelle homeostasis and activates the unfolded protein response. J Biol Chem 2011; 286: 11951–11959. 21266578 10.1074/jbc.M110.215038 PMC3069397

[bib6] 6Gallo-Ebert C, McCourt PC, Donigan M, Villasmil ML, Chen W, Pandya D et al. Arv1 lipid transporter function is conserved between pathogenic and nonpathogenic fungi. Fungal Genet Biol 2012; 49: 101–113. 22142782 10.1016/j.fgb.2011.11.006 PMC3278566

[bib7] 7Swain E, Stukey J, McDonough V, Germann M, Liu Y, Sturley SL et al. Yeast cells lacking the ARV1 gene harbor defects in sphingolipid metabolism. Complementation by human ARV1. J Biol Chem 2002; 277: 36152–36160. 12145310 10.1074/jbc.M206624200

[bib8] 8Fei W, Alfaro G, Muthusamy BP, Klaassen Z, Graham TR, Yang H et al. Genome-wide analysis of sterol-lipid storage and trafficking in *Saccharomyces cerevisiae*. Eukaryot Cell 2008; 7: 401–414. 18156287 10.1128/EC.00386-07 PMC2238164

[bib9] 9Ruggles KV, Garbarino J, Liu Y, Moon J, Schneider K, Henneberry A et al. A functional, genome-wide evaluation of liposensitive yeast identifies the "ARE2 required for viability" (ARV1) gene product as a major component of eukaryotic fatty acid resistance. J Biol Chem 2014; 289: 4417–4431. 24273168 10.1074/jbc.M113.515197 PMC3924304

[bib10] 10Villasmil ML, Nickels JT Jr. Determination of the membrane topology of Arv1 and the requirement of the ER luminal region for Arv1 function in *Saccharomyces cerevisiae*. FEMS Yeast Res 2011; 11: 524–527. 21539707 10.1111/j.1567-1364.2011.00737.x

[bib11] 11Wendland M, Willenzon S, Kocks J, Davalos-Misslitz AC, Hammerschmidt SI, Schumann K et al. Lymph node T cell homeostasis relies on steady state homing of dendritic cells. Immunity 2011; 35: 945–957. 22195748 10.1016/j.immuni.2011.10.017

[bib12] 12Ryan MJ, McLemore GR Jr, Hendrix ST. Insulin resistance and obesity in a mouse model of systemic lupus erythematosus. Hypertension 2006; 48: 988–993. 16982954 10.1161/01.HYP.0000243612.02929.df

[bib13] 13Livak KJ, Schmittgen TD. Analysis of relative gene expression data using real-time quantitative PCR and the 2(-Delta Delta C(T)) Method. Methods 2001; 25: 402–408. 11846609 10.1006/meth.2001.1262

[bib14] 14Varela GM, Antwi DA, Dhir R, Yin X, Singhal NS, Graham MJ et al. Inhibition of ADRP prevents diet-induced insulin resistance. Am J Physiol Gastrointest Liver Physiol 2008; 295: G621–G628. 18669627 10.1152/ajpgi.90204.2008 PMC2536783

[bib15] 15Ookhtens M, Baker N. Fatty acid oxidation to H2O by Ehrlich ascites carcinoma in mice. Cancer Res 1979; 39: 973–980. 427783

[bib16] 16Baker N, Morris D, Sandborg C. Blood sampling techniques for studying rapidly turning over metabolic fuels in mice. Lipids 1976; 11: 818–820. 994753 10.1007/BF02533411

[bib17] 17Virtue S, Even P, Vidal-Puig A. Below thermoneutrality, changes in activity do not drive changes in total daily energy expenditure between groups of mice. Cell Metab 2012; 16: 665–671. 23140644 10.1016/j.cmet.2012.10.008 PMC3556741

[bib18] 18Liu L, Jiang Q, Wang X, Zhang Y, Lin RC, Lam SM et al. Adipose-specific knockout of SEIPIN/BSCL2 results in progressive lipodystrophy. Diabetes 2014; 63: 2320–2331. 24622797 10.2337/db13-0729

[bib19] 19Cui X, Wang Y, Tang Y, Liu Y, Zhao L, Deng J et al. Seipin ablation in mice results in severe generalized lipodystrophy. Hum Mol Genet 2011; 20: 3022–3030. 21551454 10.1093/hmg/ddr205

[bib20] 20Chen W, Chang B, Saha P, Hartig SM, Li L, Reddy VT et al. Berardinelli-seip congenital lipodystrophy 2/seipin is a cell-autonomous regulator of lipolysis essential for adipocyte differentiation. Mol Cell Biol 2012; 32: 1099–1111. 22269949 10.1128/MCB.06465-11 PMC3295006

[bib21] 21Peterfy M, Phan J, Xu P, Reue K. Lipodystrophy in the fld mouse results from mutation of a new gene encoding a nuclear protein, lipin. Nat Genet 2001; 27: 121–124. 11138012 10.1038/83685

[bib22] 22Cortes VA, Curtis DE, Sukumaran S, Shao X, Parameswara V, Rashid S et al. Molecular mechanisms of hepatic steatosis and insulin resistance in the AGPAT2-deficient mouse model of congenital generalized lipodystrophy. Cell Metab 2009; 9: 165–176. 19187773 10.1016/j.cmet.2009.01.002 PMC2673980

[bib23] 23Moitra J, Mason MM, Olive M, Krylov D, Gavrilova O, Marcus-Samuels B et al. Life without white fat: a transgenic mouse. Genes Dev 1998; 12: 3168–3181. 9784492 10.1101/gad.12.20.3168 PMC317213

[bib24] 24Tong F, Billheimer J, Shechtman CF, Liu Y, Crooke R, Graham M et al. Decreased expression of ARV1 results in cholesterol retention in the endoplasmic reticulum and abnormal bile acid metabolism. J Biol Chem 2010; 285: 33632–33641. 20663892 10.1074/jbc.M110.165761 PMC2962461

[bib25] 25Kraemer K, Schmidt U, Fuessel S, Herr A, Wirth MP, Meye A. Microarray analyses in bladder cancer cells: inhibition of hTERT expression down-regulates EGFR. Int J Cancer 2006; 119: 1276–1284. 16615118 10.1002/ijc.21975

[bib26] 26Anderson EM, Miller P, Ilsley D, Marshall W, Khvorova A, Stein CA et al. Gene profiling study of G3139- and Bcl-2-targeting siRNAs identifies a unique G3139 molecular signature. Cancer Gene Ther 2006; 13: 406–414. 16195754 10.1038/sj.cgt.7700901

[bib27] 27Kakiuchi-Kiyota S, Koza-Taylor PH, Mantena SR, Nelms LF, Enayetallah AE, Hollingshead BD et al. Comparison of hepatic transcription profiles of locked ribonucleic acid antisense oligonucleotides: evidence of distinct pathways contributing to non-target mediated toxicity in mice. Toxicol Sci 2014; 138: 234–248. 24336348 10.1093/toxsci/kft278

[bib28] 28Bilanges B, Stokoe D. Direct comparison of the specificity of gene silencing using antisense oligonucleotides and RNAi. Biochem J 2005; 388: 573–583. 15656792 10.1042/BJ20041956 PMC1138965

