# Supplementary material for: Deletion of murine Arv1 results in a lean phenotype with increased energy expenditure
Source: Nutr Diabetes. 2015 Oct 19;5(10):e181–. doi: 10.1038/nutd.2015.32 (PMC4631934; doi:10.1038/nutd.2015.32)
Supplement: Supplementary Informations [file nutd201532x1.pdf]

	**Relative *Arv1* mRNA**
**Adrenal**	**33.48 +/- 6.02**
**Skeletal Muscle**	**22.40 +/- 4.93**
**Liver**	**6.41 +/- 1.48**
**Heart**	**5.10 +/- 2.03**
**Brain**	**4.98 +/- 2.11**
**Ovary**	**4.68 +/- 2.21**
**Kidney**	**3.98 +/- 0.39**
**Lung**	**2.15 +/- 0.60**
**Inguinal Subcutaneous WAT**	**2.11 +/- 0.21**
**Spleen**	**1.00 +/- 0.41**

Supplemental Figure 1. *Arv1* mRNA expression in female mouse tissues. *Arv1* mRNA expression was determined by quantitative RT-PCR of total RNA using B-Actin as the housekeeping gene, and normalized to the least abundant tissue (spleen). Data from technical replicates are reported as the mean +/- S.D. for each tissue.

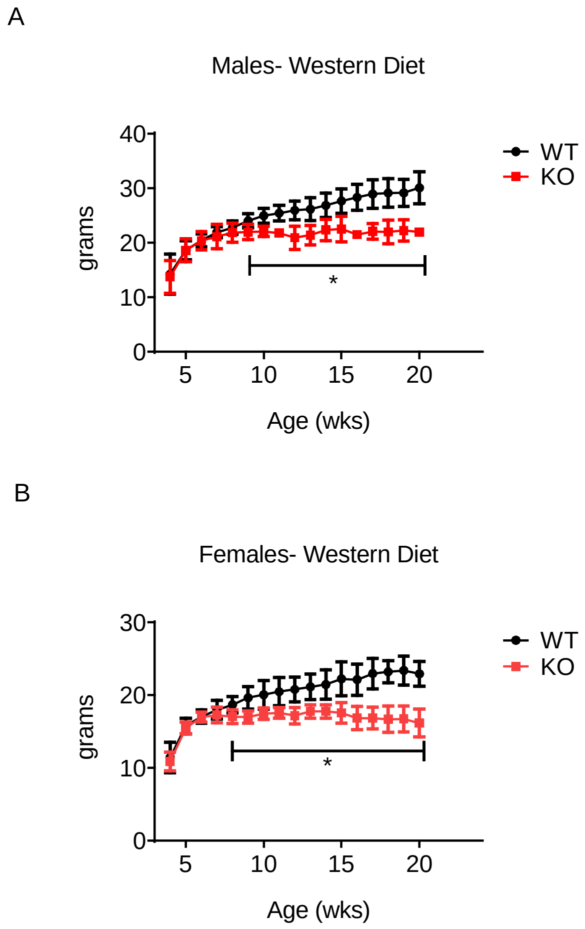

Supplemental Figure 2. Growth curves of *Arv1* KO mice fed a Western Diet- WT and *Arv1* KO were fed a western diet beginning at time of weaning and body weights were recorded weekly. The western diet contains 0.21 % cholesterol and 1 % corn oil (w/w); fat accounts for 41% of total calories (Research Diets D12079B). (**A**) Male WT (n = 4-7) and *Arv1* KO (n = 2-4) mice and (**B**) Female WT (n = 6-7) and *Arv1* KO (n = 5-10) mice.

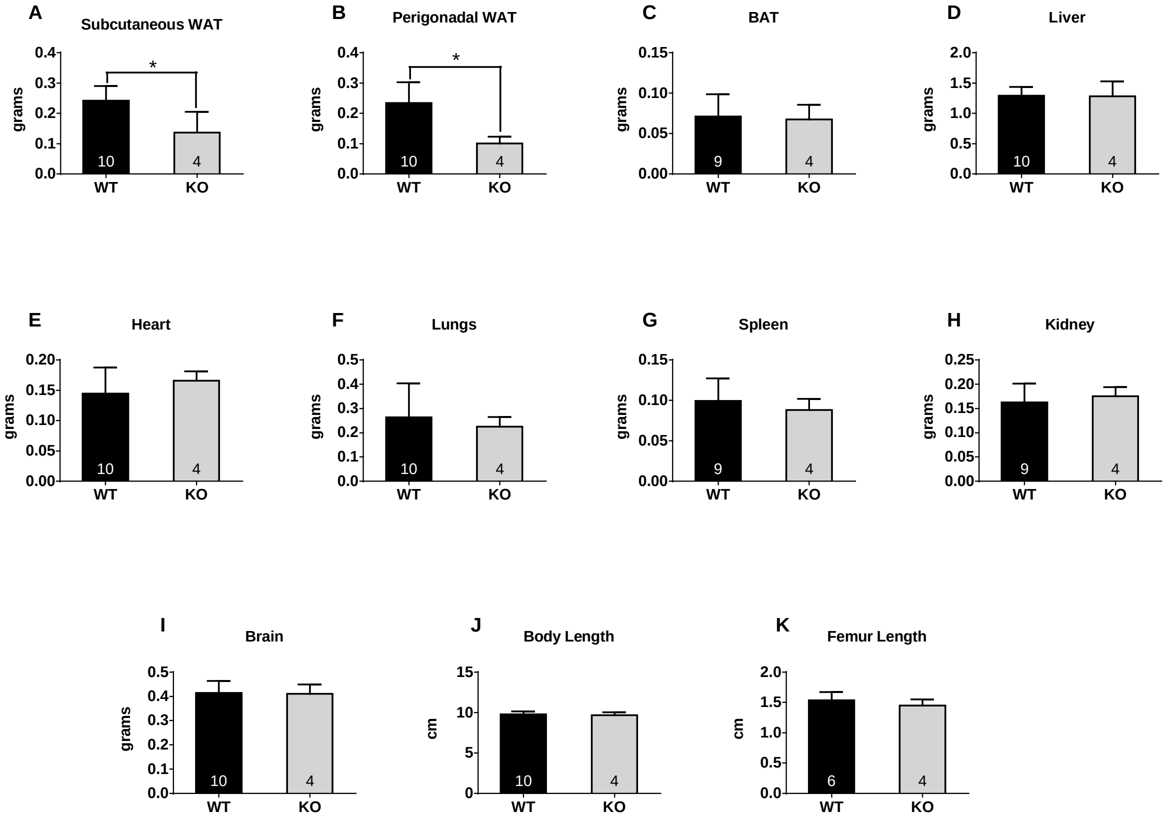

Supplemental Figure 3. Adipose and tissue weights in *male* WT and *Arv1* KO mice. Tissue weights and sizes were determined by dissection in chow fed male mice at 12 weeks of age- (**A**) subcutaneous WAT, (**B**) perigonadal WAT, (**C**) BAT, (**D**) liver, (**E**) heart, (**F**) lungs, (**G**) spleen, (**H**) kidney, (**I**) brain, (**J**) body length, and (**K**) femur length. Data is reported as the mean +/- S.D. with the "*n*" for each tissue on the bar. Comparisons were made by student's t-test and significance was assigned at $*p<0.01$.

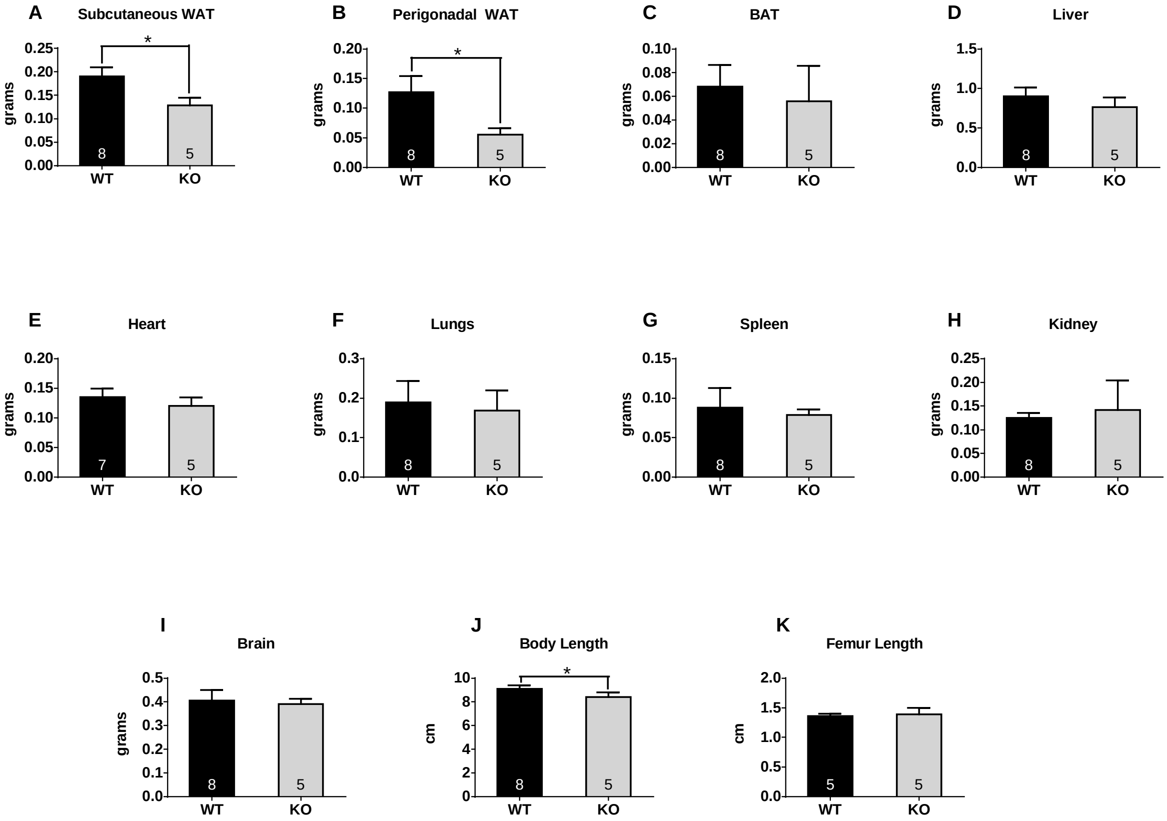

Supplemental Figure 4. Adipose and tissue weights in *female* WT and *Arv1* KO mice. Tissue weights and sizes were determined by dissection in chow fed female mice at 12 weeks of age- (**A**) subcutaneous WAT, (**B**) perigonadal WAT, (**C**) BAT, (**D**) liver, (**E**) heart, (**F**) lungs, (**G**) spleen, (**H**) kidney, (**I**) brain, (**J**) body length, and (**K**) femur length. Data is reported as the mean +/- S.D., with the "*n*" for each tissue on the bar. Comparisons were made by student's t-test and significance was assigned at *$p<0.01$.

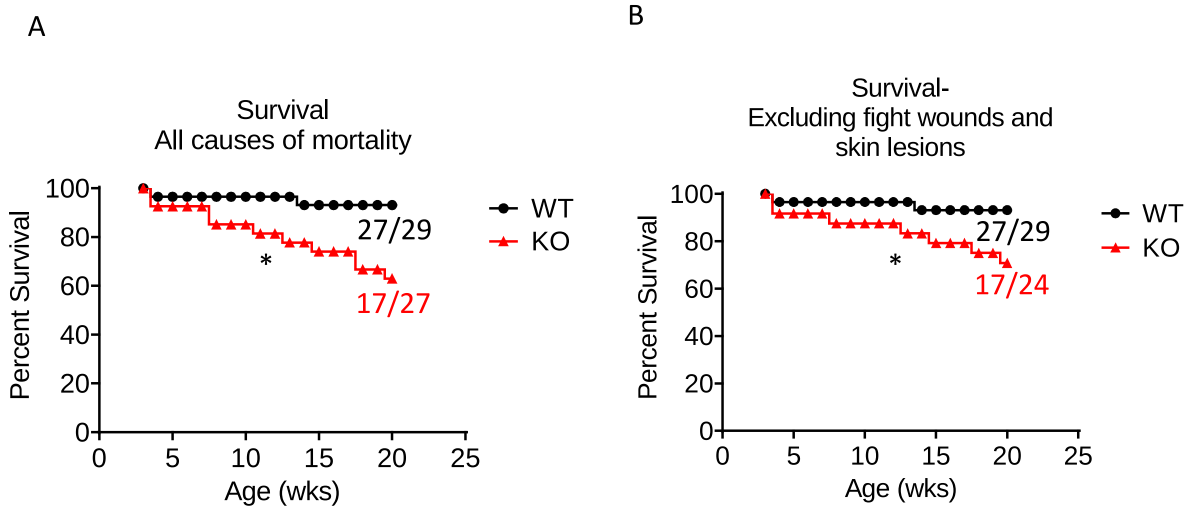

Supplemental Figure 5. Survival curves in the Arv1 KO mice. Combined survival curves for WT and *Arv1* KO mice were generated from male and female mice on both chow and western diets. (**A**) Survival curve for mice that died from all causes of mortality. (**B**) Survival curve excluding animals which died from fight wounds or were euthanized due to skin lesions.

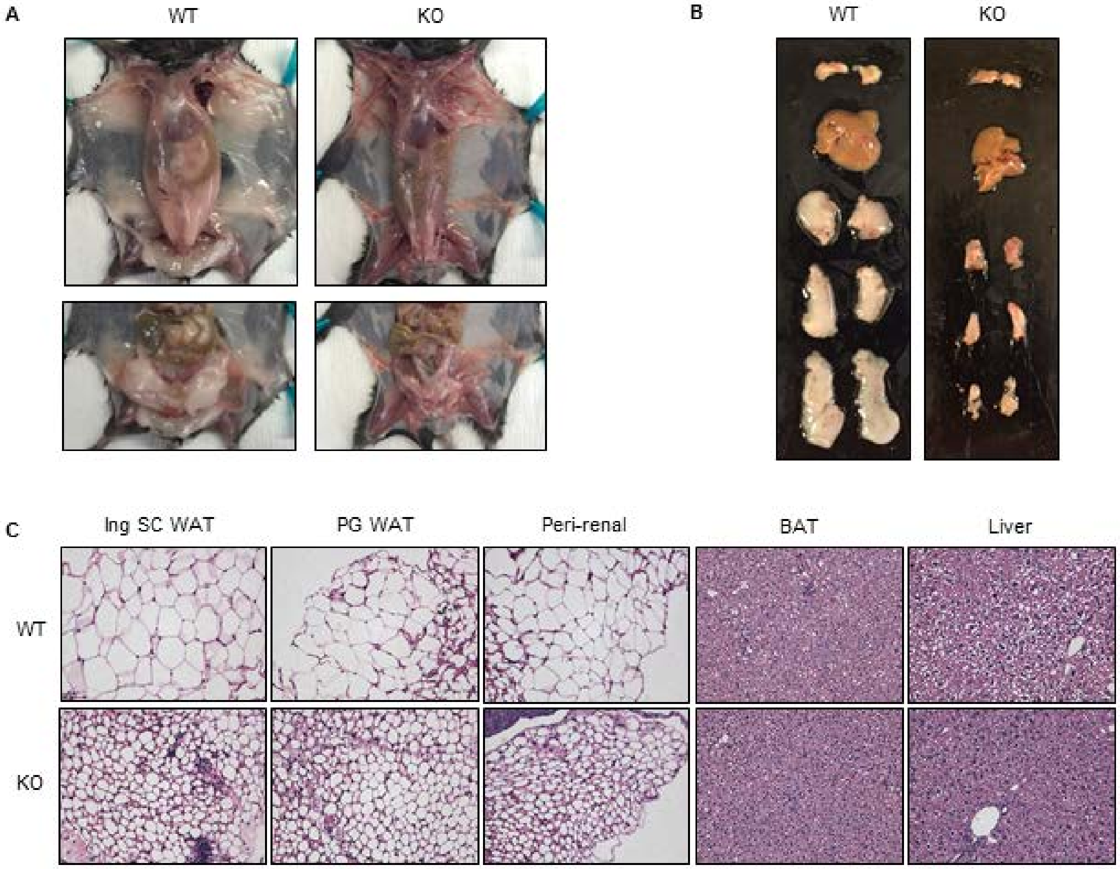

Supplemental Figure 6. Abnormal white adipose tissue morphology in female *Arv1* KO mice. (**A**) Gross appearance of littermate female WT and *Arv1* KO mice at 6 months of age showing lack of subcutaneous (SC WAT) and perigonadal (PG WAT) white adipose tissue. (**B**) Image of brown adipose tissue (BAT), liver, axillo-thoracic subcutaneous white adipose tissue (Axillo-thoracic SC WAT), inguinal subcutaneous white adipose tissue (Ing SC WAT), and perigonadal white adipose tissue (PG WAT). (**C**) Histological appearance of hematoxylin and eosin stained subcutaneous inguinal subcutaneous WAT (Ing SC WAT), perigonadal WAT (PG WAT), peri-renal WAT (PR WAT), brown fat (BAT), and liver at 400X magnification.

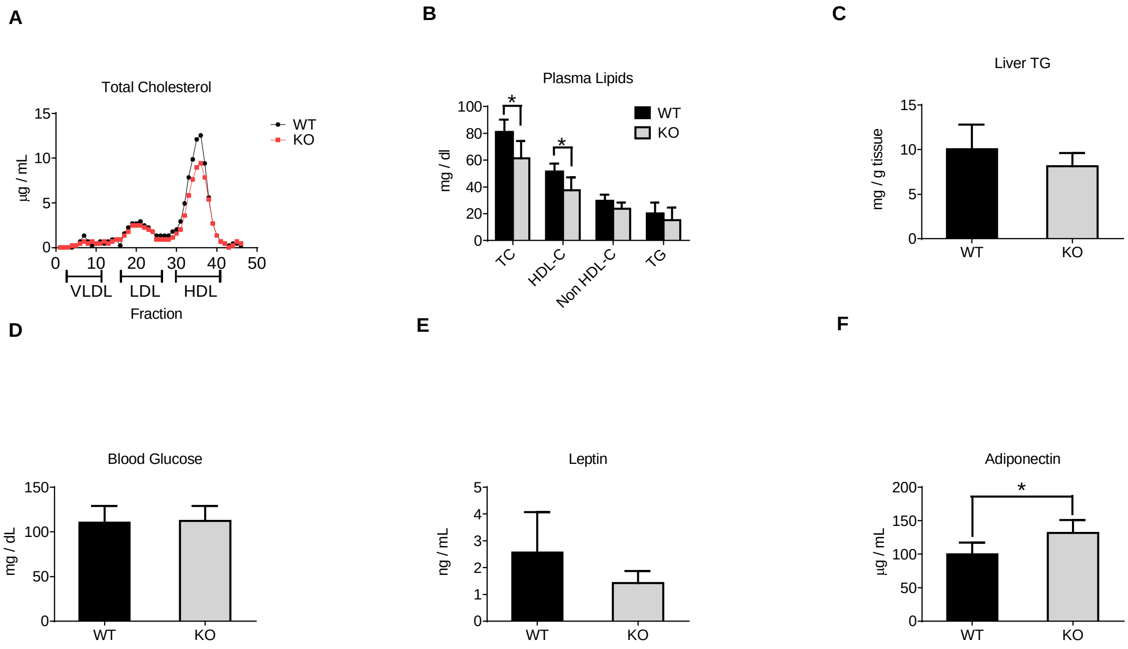

Supplemental Figure 7. Plasma lipids, glucose and adipokine levels in female *Arv1* KO mice. (**A**) Total cholesterol in lipoprotein fractions of pooled plasma from 10 week old female WT and *Arv1* KO mice fasted 4 hours. (**B**) Fasting plasma lipid levels (4 hours) were determined in chow fed female WT (n = 17) and *Arv1* KO mice (n = 9). (**C**) Fasting blood glucose (4 hours) in female WT (n = 11) and *Arv1* KO mice (n = 9). (**D**) Liver triglycerides were measured in female WT (n = 8) and *Arv1* KO mice (n = 5) at 12 weeks of age. (**E**) Plasma leptin levels in female WT (n = 8) and *Arv1* KO mice (n = 5) mice at 10 weeks of age. (**F**) Plasma adiponectin levels male WT (n = 10) and *Arv1* KO mice (n = 10) mice at 10 weeks of age. All values are reported as the mean +/- S.D. (* p< 0.05).

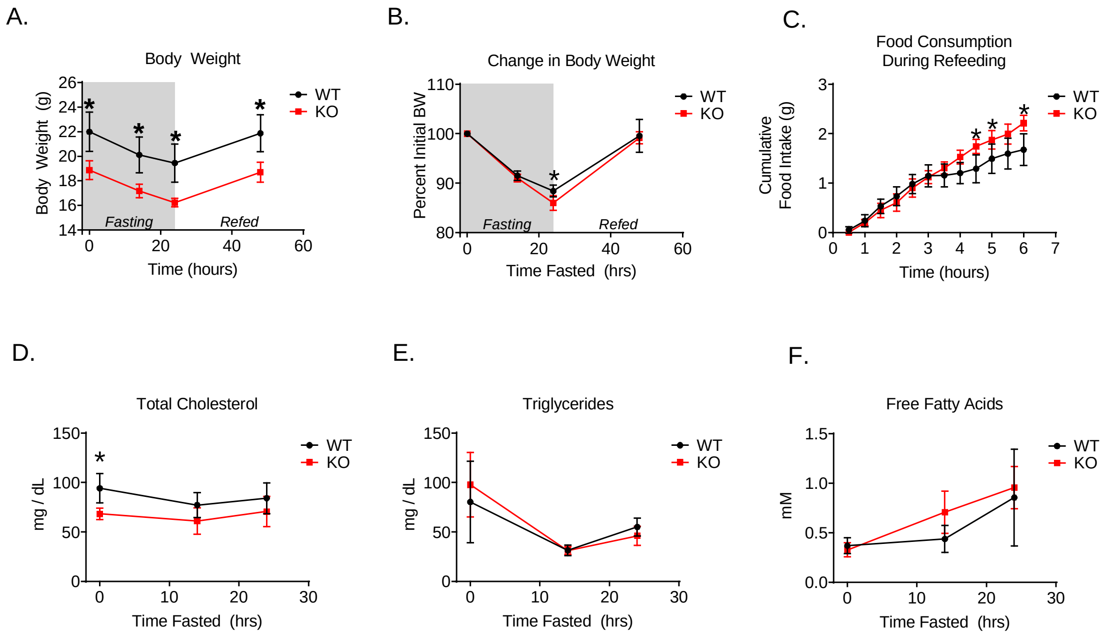

Supplemental Figure 8. Fasting challenge in female *Arv1* KO mice. Female wild type (WT) (n = 7) and Arv1 KO mice (n = 3), 2-4 months of age, were subjected to a 24 hour fasting challenge, followed by a 24 hour refeeding period. Plasma was collected at 0, 14, and 24 hours of fasting for plasma lipid measurements. Body weight was measured at 0, 14, and 24 hours of fasting, and then again at 48 hours (24 hours of refeeding). Food consumption was measured every half hour during the first 6 hours of the refeeding period. **(A)** Body weights, **(B)** body weight as percent of the initial weight, **(C)** cumulative food consumption during refeeding, **(D)** total cholesterol, **(E)** plasma triglycerides, and **(F)** plasma free fatty acids are shown. All values are presented as the mean +/- S.D. for each time point, where an asterisk indicates $p < 0.05$.
